# Differences in need for and access to eye health services between older people with and without disability: A cross-sectional survey in four districts of northern Uganda

**DOI:** 10.1371/journal.pgph.0003645

**Published:** 2024-09-10

**Authors:** Emma Jolley, Calum Davey, Stevens Bechange, Gladys Atto, Denis Erima, Ambrose Otim, Juliet Sentongo, Anthony Wani, Tesfaye Adera, Moses Kasadhakawo, Hannah Kuper

**Affiliations:** 1 Research Team, Sightsavers, Haywards Heath, United Kingdom; 2 London School of Hygiene and Tropical Medicine, London, United Kingdom; 3 Sightsavers Uganda, Kampala, Uganda; 4 Ophthalmology Department, Moroto Regional Referral Hospital, Moroto, Uganda; 5 Ophthalmology Department, Masaka Regional Referral Hospital, Masaka, Uganda; 6 Ophthalmology Department, Kiruddu National Referral Hospital, Kampala, Uganda; 7 Ophthalmology Department, Mulago National Referral Hospital, Kampala, Uganda; SRMIST: SRM Institute of Science and Technology (Deemed to be University), INDIA

## Abstract

Eye health and disability are both common among older people, and it is important to understand how disability relates to visual health status and access to services. While people with disabilities face barriers to accessing health services, few studies have measured participants’ functional status in domains other than vision and little evidence exists on how disability impacts eye health services access. This paper describes how visual impairment and access to eye health services differ between people aged 50 years and above with and without disability in Karamoja, Uganda, and explores the factors driving that difference. This was a cross-sectional survey among individuals aged 50 years and above. A standardised eye health survey was conducted, with additional questions on personal and health characteristics. Ophthalmologists conducted a vision examination, and recorded participants’ self-reported functional difficulties using the Washington Group Short Set Enhanced. Descriptive analyses were conducted using Stata, and multivariate models constructed to explore relationships. 21.7% of respondents self-reported some sort of functional difficulty. Twenty-five percent of individuals with a non-visual functional difficulty are also blind, and a further 29% experience a lower level of VI. In a multivariate model, blindness was associated with self-reported difficulties seeing, but not any other type of difficulty. Blindness was also associated with age, not being married, and living in a smaller household. Access to cataract surgery was associated with non-visual functional difficulties, male gender, and having a regular household income. This study confirms that in the study area, disability and visual impairment are common among people aged 50 years and above, access to eye health services is low, and self-reported functional difficulties are not associated with lower access to services.

## Introduction

Robust quantitative evidence about the health of older people with disabilities in sub-Saharan Africa is scarce [1]. While on a global level older age is well understood to be the period of life when individuals experience the highest rates of mortality, morbidity, and disability [2], there is little data exploring the situation of older individuals in African countries [3]. The data that does exist suggests that the health conditions that affect older people in sub-Saharan Africa–hypertension, musculoskeletal disease, visual and hearing impairments, and depression–continue to be neglected, with insufficient resources, little data, few healthcare specialists, and no or outdated policies available to support effective service provision [4,5]. Data does not tend to explore how the situation differs by disability status, but global evidence suggests that people with disabilities tend to have greater unmet health needs than people without disabilities [6,7]. This is an important issue as estimates from studies across different countries in sub-Saharan Africa suggest that the prevalence of disability is high among the over 50s and increases sharply with advanced age. For example, recent surveys from South Africa and Uganda suggest the prevalence of disability among 50 to 54 years olds ranges from between 9.0% and 32.9%, increasing to between 56.6% and 68.1% among the over 80s [8,9]. Moreover, the proportion of older adults in the region is growing faster than elsewhere and so it is projected that by 2050 there may be as many as 100 million older people living with disabilities [5,10].

Individuals with disability experience the same health needs as others but they may also have additional needs linked to their impairment [11]. Research shows that disability intersects with other characteristics, e.g. age, gender, socio-economic status, contributing to worse outcomes among people with multiple markers of disadvantage [6,12]. Furthermore, health services are often designed and delivered in a way that do not adequately accommodate the needs of people with different functional difficulties, shaping how individuals are able to navigate care pathways and resulting in a multitude of barriers to accessing services [6,12]. For example, individuals with difficulties hearing or communicating may be less likely to receive information in an accessible format about health conditions, or available services [12–14]. Difficulties in these domains, or those of cognition or remembering, may also make communication with health workers more difficult, particularly for individuals without support from families or caregivers [12–14]. Communication with healthcare workers may be further undermined by stigma and lack of disability training for the professional. Many people with disabilities report not accessing care due to past experiences with–or anticipation of–negative attitudes among healthcare workers [12–14]. Difficulties with vision or mobility may mean that accessing services located further away from home is more difficult, requiring greater financial resources or social support [12,13]. Poorly designed or maintained buildings may be harder for some individuals to enter, and lack of seating or inaccessible toilets may make the long waiting times more difficult or uncomfortable [12,14]. People who experience mental health difficulties such as anxiety or depression may feel particularly nervous around health workers, particularly at the thought of an intervention like surgery [15]. In some cultures, myths persist around clinical and surgical procedures that can deter many individuals from accessing services, particularly those already uncomfortable with leaving their home environment [12,16].

The majority of visual impairment (VI) is concentrated among older people with more than 80% of blindness found among people aged 50 years and above in most low- and middle-income countries [17,18]. The most common causes of VI (cataract, unaddressed refractive error, and trachoma) are simple to identify and treat when people have access to good quality services [17]. However, uptake of eye services is often poor, especially in sub-Saharan Africa. This situation may be even worse for people with disabilities through the pathways described above. This is important not only because over half of older people have disabilities, but also because individuals with VI may be more likely to experience difficulties in other domains due to their condition [17].

The aim of this paper is to describe how visual impairment and access to eye health services differ between people aged 50 years and above with and without disability in Karamoja, northern Uganda, and to explore how other factors drive that difference. While people with disabilities are recognised as facing multiple barriers to accessing health services, few studies have sought to measure participants’ disability or functional status in domains other than vision and thus little empirical evidence exists on how difficulties functioning in different domains may impact on access to eye health services. Given how common both eye health and disability are among older people, it is important to explore how they relate, and to explore differences in visual health status and access to services.

## Materials and methods

This cross-sectional survey followed standard rapid assessment of avoidable blindness (RAAB) methodology [19], with additional questions added to elicit further information regarding individuals’ personal and health circumstances. Data was collected between February 13^th^ and 26^th^ 2023.

### Study setting

Karamoja is a sub-region of northern Uganda with a 2022 population of approximately 1,245,600 [20]. Because of ongoing security issues linked to cattle raiding in parts of the sub-region, the study was conducted in four of the nine districts deemed to be safe: Moroto, Napak, Nabilatuk and Nakapiripirit. An ophthalmologist has been stationed at Moroto Regional Referral hospital since 2019 and ophthalmic clinical officers based in each district work with hospital staff to create awareness in communities, identify people in need of services, and provide services, with a particular emphasis on cataract and trachoma surgeries.

### Sampling

The sample size was powered to measure the difference in prevalence of severe VI between people with and without disabilities aged 50 years and above, as this is where the majority of VI is found [18,21]. From similar past surveys, we assumed a disability prevalence of 10%, and the prevalence of severe VI among people with disabilities to be double that among people without disabilities [22]. We allowed 95% confidence intervals, 20% precision, 10% non-response, and 50% design effect. The required sample size was 3,427, or 69 clusters of 50 persons, yielding a total sample size of 3,450.

The study population was people aged 50 years and above who have been resident in the study area for at least six months. Two stage sampling was used: villages were selected using probability proportional to size, and individuals within villages were selected using modified compact segment sampling. The study team enumerated all eligible individuals, including those temporarily absent, and those present underwent study procedures. Basic data about absent participants was collected from their family members or neighbours.

### Study procedures

Data were collected by five teams, each with an ophthalmologist and ophthalmic clinical officer who participated in a five-day training and passed an intra-observer variation test before data collection. Eligible consenting individuals underwent a vision examination and were asked personal and health, questions. All data were collected using an app designed on CommCare on a tablet [23]. Locations of villages were recorded using global positioning system (GPS) coordinates.

### Vision examination

The standard RAAB protocol is described elsewhere [19]. In brief, participants were asked about their spectacle use, both eyes had presenting (with available correction) visual acuity (PVA) measured, and each lens was examined in a darkened room to identify signs of unoperated or operated cataract. All eyes with PVA<6/12 had their best corrected visual acuity (BCVA) measured using a pinhole. Ophthalmologists assessed all eyelids for signs of trachoma infection or surgery. Ophthalmologists assessed the primary cause of VI of each eye with PVA <6/12. Every eye with PVA <6/12 where the principal cause could not be attributed to unaddressed refractive error, unoperated cataract, or corneal scarring was dilated with a short acting mydriatic and was examined with a direct ophthalmoscope in a darkened room.

Minor ocular conditions such as conjunctivitis were treated by the team. Other conditions were referred to the nearest appropriate health centre or hospital.

Outcome variables reported in this paper were derived from the visual examination. Categories of VI were assigned using the WHO ICD-11 definitions [24]:

- **Blindness:** PVA<3/60 in the better eye.
- **Severe VI**: PVA ≥3/60 but <6/60 in the better eye.
- **Moderate VI**: PVA ≥6/60 but <6/18 in the better eye.
- **Early VI**: PVA ≥6/18 but <6/12 in the better eye.
- **Normal vision:** PVA of 6/12 or better.

Access to eye health services was defined by the number of people identified as having accessed a service, as a proportion of all those who required it, as follows:

- **Cataract surgery:** individuals with operated cataract in one or both eyes, as a proportion of people with operated plus unoperated cataract.
- **Refractive error services:** individuals with spectacles whose corrected vision is ≥6/12, as a proportion of people with corrected vision plus unaddressed refractive error (individuals with PVA<6/12 in the better eye who do not have distance correction but who improve to BCVA ≥6/12).
- **Trachoma surgery:** individuals with operated trachoma in one or both eyes, as a proportion of people with operated trachoma plus unoperated trachoma, regardless of visual acuity.

### Personal and health characteristics

Disability was measured through self-reported functional difficulties in eight domains based on the Washington Group’s ‘*Short Set–Enhanced’* question set (i.e. seeing, hearing, walking, communication, remembering/ concentrating, self-care, anxiety, and depression) [25]. Response categories were non-binary, allowing for analysis of severity as well as type. We created one measure of functional difficulties and one of mental health difficulties:

1. Functional difficulties: a trichotomous variable with individuals reporting *‘a lot of difficulty’* or *‘cannot do at all’* in a) the seeing domain (these individuals may also have reported difficulties in other domains), b) in domains other than seeing (*hearing*, *walking*, *communication*, *remembering/ concentrating*, *and self-care*), or c) no difficulty in any domain.
2. Mental health difficulties: a binary variable with individuals reporting either *anxiety* or *depression* on a ‘daily’ basis, with the level of feelings being ‘*a lot’*, considered as having mental health difficulties.

Poverty: we asked whether the household has a regular income or not, resulting in a binary variable.

Social support: we included three measures of social support: marital status, the number of people living in the individual’s immediate household, and the number of children living in proximity to the individual.

Health services: we collected the cluster GPS coordinates during the survey and identified all health facilities, with and without ophthalmologists using Google Maps imagery. Distances between clusters and health facilities were calculated in ArcGIS Pro 3.1 [26], using the road network data from Open Street Map and expressed in units of 10km, and then exported into Stata where they were linked to individual records using the cluster id. Two quantitative variables were generated: “Distance from health centre, 10km from centre with ophthalmologist” and “Distance from health centre, 10km from any centre. Participants were also asked whether they accessed health care the last time they needed it (yes/ no).

### Statistical analysis

Statistical analyses were conducted in Stata 15 [27]. Descriptive analyses were conducted by cross-tabulating outcome variables by functional difficulties and mental health difficulties. Relationships were measured using prevalence ratios (PRs) calculated using Poisson regression with robust standard errors that accounted for sample clustering and were adjusted for age and gender [28].

Variables found to have a relationship with the outcome (95% CIs not including 1.00) were added into a multivariable Poisson regression model with robust standard errors along with functional difficulties and mental health difficulties to estimate adjusted prevalence rations (APRs).

### Ethics

This study was reviewed and approved by institutional review boards at the London School of Hygiene and Tropical Medicine (Ref: 28285), Mulago Hospital Research Ethics Committee (Ref: MHREC-2022-83), and Uganda National Council for Science and Technology (Ref: SS1558ES). All participants underwent an accessible and meaningful informed consent process and were provided with UGX 5,000 (approx. GBP1.00) compensation for their time. Written consent was obtained from all participants, except those illiterate or unable to sign their name for any other reason, and those participants provided a thumbprint, witnessed by an independent observer.

Additional information regarding the ethical, cultural, and scientific considerations specific to inclusivity in global research is included in the (S1 Checklist).

## Results

Of the 3,450 people sampled by the study, 3,159 were enrolled (response rate 91.6%) of whom 2,502 (65.0%) were female and the mean age was 66 years.

### Participant characteristics

The most commonly reported functional difficulty was seeing, followed by walking, hearing, self-care, remembering/ concentrating, and communicating, S1 Table. Combining all six domains, 352 people (11.1%) reported a functional difficulty. 209 (6.6%) self-reported difficulty seeing, 143 (4.5%) reported a functional difficulty in one or more of the other five domains. Three hundred and eighty-three people (11.0%) experienced a lot of anxiety on a daily basis, and 195 people (5.4%) experienced a lot of depression on a daily basis. Overall, 438 people (12.5%) experienced a mental health difficulty on a daily basis.

In total, 686 people (21.7%) reported a functional difficulty, and/ or mental health difficulties. Excluding difficulties seeing, this fell to 477 people (15.1%), 70.6% of whom reported difficulties in more than one domain. After adjusting for age, difficulties in all domains were more common among women than men.

People with seeing and mental health difficulties were older and more likely to be female than those with other functional difficulties, who in turn were older than people with no functional difficulties, Table 1. People with difficulties seeing were most likely to live in households with a regular income. The majority of people with difficulties seeing were widowed, whereas the majority of all others were married. People with no functional difficulties were most likely to have more than five children living nearby and to live in larger households. People with other functional difficulties and mental health difficulties were less likely to have accessed healthcare the last time they required it. People with difficulties seeing lived the greatest mean distance from any health facility. People with mental health difficulties lived closer to any health facility than people without.

**Table 1:** Participant characteristics by disability: Seeing difficulties, other functional difficulties, and mental health difficulties.

		People with self-reported difficulties seeing (n = 209)	People with self-reported other functional difficulties (not seeing) (n = 143)	People with no self-reported functional difficulties (n = 2,807)	People with self-reported mental health difficulties (n = 438)	People with no mental health difficulties (n = 2,712)
		%	%	%	%	%
Age group	50–59	9.6	21.7	33.2	24.2	32.2
	60–69	24.4	20.3	29.7	27.4	29.2
	70–79	29.2	25.9	23.3	30.4	22.7
	80+	36.8	32.2	13.8	18.0	15.9
Gender	Female	73.7	64.3	64.3	74.4	63.4
Poverty: household regular income	Regular income	39.2	33.6	26.5	36.1	26.3
Marital status:	Married	39.2	51.1	58.4	49.3	58.0
	Widowed	55.0	44.1	37.3	45.7	37.7
	Separated or never married	5.7	4.9	4.2	5.0	4.3
Number of children living nearby	5+	38.3	44.8	64.6	42.5	65.1
	1–4	47.4	43.4	31.4	46.4	30.8
	0	14.4	11.9	4.1	11.2	4.1
Size of immediate household	Fewer than 5	31.4	37.3	23.2	28.9	23.7
Distance from health centre	10 Km from centre with ophthalmologist	Mean 5.8 (sd 4.2)	Mean 5.8 (sd 3.2)	Mean 5.9 (sd 3.6)	Mean 6.3 (sd 3.5)	Mean 5.8 (sd 3.6)
	10 Km from any centre	Mean 1.7 (sd 1.5)	Mean 1.3 (sd 1.1)	Mean 2.0 (sd 2.2)	Mean 1.4 (sd 0.7)	Mean 2.1 (sd 2.2)
Did not access healthcare last time it was needed	Did not access healthcare	14.8	21.7	9.8	19.4	9.2

sd indicates standard deviation.

### Eye health and disability

People reporting “a lot” of difficulties seeing or “cannot see at all” were the most likely to have VI. However, more than half the blind people and more than 80% of people with severe VI reported only some or no difficulties seeing, Table 2. Consequently, 5.7% people reporting other, non-visual, functional difficulties were found to be blind, as were 2.6% of people reporting no functional difficulties.

**Table 2:** Relationship between self-reported difficulties seeing, and clinically diagnosed VI.

	No difficulty (n = 2,237)	Some difficulties (n = 713)	A lot of difficulties (n = 154)	Cannot do at all (n = 55)
	%	%	%	%
Normal vision	83.0	15.9	1.1	0.04
Early VI	59.7	35.11	5.3	0.0
Moderate VI	40.2	43.0	15.9	0.9
Severe VI	38.4	45.6	16.0	0.0
Blind	19.4	32.1	22.5	26.0

The prevalence of blindness was higher among people self-reporting difficulties seeing than people with other self-reported functional difficulties, and no functional difficulties, Table 3. The prevalence of severe and moderate VI was also higher among people self-reporting difficulties seeing than people with no self-reported difficulties, but there was no observable difference with people self-reporting difficulties in other domains. There was no observable difference in early VI by self-reported functional difficulty. Blindness, severe, and early VI did not differ by mental health difficulties, but moderate VI was more likely among people reporting mental health difficulties.

People with other functional difficulties were more likely to have an operated cataract than people self-reporting difficulties seeing. There did not appear to be any difference in access to trachoma surgery by functional difficulty status. There was no observable difference in cataract or trachoma surgery by self-reported mental health difficulty status.

**Table 3:** Visual impairment and access to services by disability (difficulties seeing, other functional difficulties, mental health difficulties)—confidence intervals adjusted for clustering.

	People with self-reported difficulties seeing (n = 209)		People with self-reported other, non-visual, functional difficulties (n = 143)		People without self-reported functional difficulties (n = 2,807)		People with self-reported mental health difficulties (n = 438)		People without mental health difficulties (n = 2,721)	
**Prevalence of VI**	**%**	**95% CI**	**%**	**95% CI**	**%**	**95% CI**	**%**	**95% CI**	**%**	**95% CI**
Blind	43.1	31.8–55.1	5.7	2.6–11.8	2.6	2.0–3.4	7.4	5.1–10.5	4.5	3.5–5.7
Severe VI	9.5	6.1–14.5	3.0	1.1–8.4	2.7	2.1–3.5	2.9	1.8–4.5	3.1	2.4–3.9
Moderate VI	26.7	18.1–37.5	16.5	11.3–23.6	6.6	5.6–7.6	12.3	9.0–16.5	7.4	6.4–8.6
Early VI	8.0	4.3–14.4	9.6	5.3–16.7	7.8	6.7–9.0	9.9	7.0–13.9	7.5	6.5–8.7
**Access to services:**										
Cataract surgery	18.7	9.5–33.6	57.9	34.5–78.2	28.2	19.0–39.6	18.7	8.0–37.8	30.6	21.6–41.4
Trachoma surgery	62.6	29.9–86.8	64.1	13.9–95.2	49.9	31.6–68.2	49.0	14.5–84.5	52.9	37.4–67.7

Accounting for gender and age, blind people were more likely to self-report difficulties seeing, but not other functional difficulties, Table 4. In a multivariate model, being blind was associated with self-reported difficulties seeing, age, not being married, and living in a smaller household.

**Table 4:** Associations between visual impairment and a range of factors.

Factors	Categories	Models adjusted for age and gender only				Multivariate models			
		Blind		Any VI		Blind		Any VI	
		PR	95%CI	PR	95%CI	APR	95%CI	APR	95%CI
Functional difficulties (ref no difficulties)	Seeing	8.06	5.47–11.87***	2.25	1.95–2.59***	8.10	5.69–11.54***	2.27	2.02–2.56***
	Other	1.37	0.68–2.77	1.20	0.96–1.50	1.32	0.67–2.60	1.18	0.98–1.42
Mental health difficulties (ref no difficulties)		1.54	1.10–2.16*	1.31	1.16–1.49***	1.03	0.76–1.39	1.04	0.94–1.15
Age (ref 50–59) ^†^	60–69	3.79	2.10–6.82***	3.13	2.40–4.08***	2.95	1.69–5.15***	2.88	2.23–3.71***
	70–79	4.89	2.80–8.53***	5.81	4.59–7.35***	3.50	2.13–5.74***	5.04	3.99–6.36***
	80+	17.2	9.9–29.9***	9.37	7.32–12.01***	8.62	5.10–14.54***	7.17	5.63–9.13***
Gender (ref male) ^‡^	Female	1.55	1.11–2.16*	1.04	0.92–1.18	0.97	0.72–1.33		
Poverty (ref no regular income)	Regular income	0.60	0.41–0.87**	1.11	0.98–1.25	0.60	0.34–1.04		
Marital status (ref married)	Widowed, never married or separated	1.53	1.18–1.98**	1.31	1.19–1.45***	1.38	1.11–1.72**	1.19	1.08–1.30***
Children living near you (ref 5+ children)	<5	1.62	1.28–2.07***	1.31	1.22–1.41***	1.09	0.86–1.39	1.09	1.02–1.18*
Household size (ref 5+)	<5	1.90	1.47–2.45***	1.27	1.16–1.39***	1.73	1.38–2.17***	1.21	1.10–1.33***
Distance from health centre with ophthalmologist (per 10 km)	10 km	0.93	0.89–0.97***	0.99	0.97–1.00	0.97	0.92–1.02		
Distance from any health centre (per 10 km)	10 km	0.99	0.93–1.05	0.97	0.95–1.00				
Accessed healthcare last time it was needed (ref yes)	No	1.17	0.79–1.72	1.03	0.89–1.20				

* p<0.05

** p<0.01

*** p<0.001

^†^ adjusted for gender only

^‡^ adjusted for age only.

People with any VI including blindness were more likely to report difficulty seeing, but not other functional difficulties. In a multivariate model, having any level of VI was associated with self-reported difficulties seeing, age, not being married, fewer children living nearby, and a smaller household size.

Self-reporting other functional difficulties was associated with a greater likelihood of having an operated cataract, in both the age and gender adjusted models, and in the fully adjusted multivariate model, Table 5. There was no association with seeing or mental health difficulties and cataract operated status, and no type of functional difficulty was associated with trachoma operated status.

**Table 5:** Associations between access to eye health services and a range of factors.

Factors	Categories	Models adjusted for age and gender only				Multivariate models			
		Operated cataract		Operated trachoma		Operated cataract		Operated trachoma	
		PR	95%CI	PR	95%CI	APR	95%CI	APR	95%CI
Functional difficulties (ref no difficulties)	Seeing	0.81	0.54–1.21	1.33	0.89–1.98	0.71	0.47–1.05		
	Other	1.59	1.20–2.10**	1.47	0.79–2.75	1.37	1.01–1.86*		
Mental health difficulties (ref no difficulties)		0.77	0.57–1.04	1.15	0.77–1.72				
Age (ref 50–59) ^†^	60–69	1.20	0.49–2.92	1.26	0.63–2.53	1.31	0.60–2.88	1.21	0.64–2.27
	70–79	1.91	0.86–4.24	1.47	0.80–2.70	1.75	0.86–3.56	1.23	0.71–2.14
	80+	1.76	0.75–4.14	1.24	0.64–2.38	1.58	0.73–3.42	1.09	0.59–2.00
Gender (ref male) ^‡^	Female	0.70	0.54–0.90*	1.06	0.67–1.68	0.78	0.61–0.99*		
Poverty (ref no regular income)	Regular income	2.03	1.51–2.73***	1.69	1.25–2.27**	1.71	1.09–2.69*	1.59	1.02–2.49*
Marital status (ref married)	Widowed, never married or separated	1.04	0.83–1.29	1.41	1.04–1.91*			1.29	0.93–1.78
Children living near you (ref 5+ children)	<5	1.07	0.90–1.27	1.06	0.84–1.33				
Household size (ref 5+)	<5	1.07	0.79–1.45	0.87	0.54–1.38				
Distance from health centre with ophthalmologist (per 10 km)	10 km	1.06	1.00–1.11*	1.05	1.00–1.11*	1.03	0.95–1.12	1.00	0.94–1.07
Distance from any health centre (per 10 km)	10 km	0.92	0.86–0.99*	0.93	0.78–1.10	0.92	0.83–1.02		
Accessed healthcare last time it was needed (ref yes)	No	0.74	0.48–1.13	1.17	0.80–1.71				

* p<0.05

** p<0.01

*** p<0.001

^†^ adjusted for gender only

^‡^ adjusted for age only.

In a multivariate model having an operated cataract was also associated with male gender and having a regular household income. Only the having a regular household income was associated with operated trachoma in the multivariate model.

Sensitivity analyses (S2 Table) explored the impact of a scenario where all individuals with severe VI or blindness reported difficulties seeing, on the relationship with access outcomes (not eye health status due to collinearity). This multivariate analysis revealed that operated cataract was less likely among individuals with difficulties seeing (ARR 0.65, 95%CI 0.47–0.91), and was not associated with other functional difficulties (ARR 1.24, 95%CI 0.87–1.76). Other variables in the model remained broadly unchanged. No differences were observed in the operated trachoma model.

## Discussion

This study found a high proportion of people (21.7%) aged 50 years and above in the Karamoja region self-reporting some type of functional or mental health difficulty, around half of whom (10.7%) reported difficulties in more than one domain of functioning. Twenty-five percent of individuals with a non-visual functional difficulty are also blind, and a further 29% experience a lower level of VI. Although the age groups are not directly comparable, these results echo those reported by national surveys, where 40% of over 65s and 57% of over 80s were reported to have a disability [29,30]. Despite the challenges measuring disability in this study, the estimates of functional difficulties are high and support previous suggestions that older people are highly likely to live with multimorbidity and provides context that eye health services–along with others–that serve the needs of older people must be designed with their complex health and functioning needs in mind [31].

The study found no differences in VI or having been operated for trachoma between people with and without self-reported functional difficulties in domains other than seeing, or between those with and without mental health difficulties. Multivariate analysis revealed that people with self-reported difficulties in domains other than seeing were more likely to have an operated cataract than people with no functional difficulties. Although we are not aware of other studies exploring cataract surgical status by disability, these findings run contrary to the majority of evidence which tends to suggest individuals with disabilities have worse health status and access to health services than others [11].

Access to cataract surgery in the study area is very low overall–only 35.4% of people requiring cataract surgery having received it, and the study location, Karamoja is often described as one of the least developed parts of Uganda with levels of poverty at 60% [32]. Some evidence suggests that differences between people with and without disabilities only become apparent in places with significant development, when people with disabilities and other characteristics of disadvantage, fail to access the opportunities available, and fall behind [33]. It is therefore important that as the health system develops and services expands, consideration is paid to equity of access for people with disabilities to protect any level of equity currently observed.

In addition to having other functional difficulties, people with operated cataract were also more likely to be male and live in a household with a regular income; the latter also being associated with operated trachoma. This is in line with existing evidence that shows that men are more likely to have been operated and tend to have greater levels of access to cataract and trachoma surgery than women, often because of gendered social roles that determine decision making processes and access to household resources [34–36]. Although past studies frequently identify ‘cost’ as a barrier to cataract surgery, some suggest it may mask the more complex issue of family decision making processes, gender related roles which may also combine with perception of need for surgery and characteristics of local services to determine an individual’s willingness to pay for a surgery and availability of time to go for surgery [37,38]. Provision of good quality services, and counselling for family members along with the individuals have been suggested as ways to overcome this barrier, although their effectiveness has not yet been evaluated [35,37].

Factors associated with blindness and any VI were self-reported difficulties seeing, increasing age, not being married, and living in a smaller household. People with any VI were also more likely to have fewer children living nearby. These are mostly measures of social support and indicate that lack of spousal or familial support in the household or nearby may be associated with a greater likelihood of unaddressed VI, particularly among the elderly. This concurs with a number of studies exploring barriers to eye health services across Africa which suggest that not only do many individuals rely on family members–primarily spouses and children–across different stages of the health seeking journey, but family responsibilities also act as an incentive to seek care and regain visual function [16,38]. Health service providers should consider the importance of social support when planning services, particularly for older people. Social support is not routinely captured in eye health surveys such as RAABs, but a simple standardised measure such as marital status could help indicate groups of individuals at risk of not accessing services. The WHO has published various reports that advocate for the reorientation of primary care towards individuals’ needs, however, the practical application in resource-constrained environments remains unfulfilled [5,6,39]. The development of locally appropriate and cost-effective strategies to achieve this requires collaboration between policy makers, health care workers, and researchers at international and local levels. Evidence suggests that training healthcare workers on disability, including through the involvement of people with disabilities, can be effective in improving a range of outcomes including their knowledge, competence, confidence and self-efficacy [40].

We observed significant discrepancies between clinically assessed visual impairment and self-reported difficulties related to seeing, with many blind and severely visually impaired individuals reporting some or no difficulties seeing. This has been observed elsewhere: a study from Cameroon reported 99% sensitivity and 30% specificity in the visual domain and similar discrepancies in the hearing and mobility domains [41]. We did not clinically assess non-vision domains in this study, but it is possible that by using the self-reported measures we underestimated the prevalence of clinically significant impairments in those domains. The authors suggest agreement may be higher if the definition of disability using the self-reported questions is expanded to include ‘some difficulty’ as well as those reporting ‘a lot’ or ‘cannot do at all’ [41,42]. Self-reported difficulties seeing may vary between individuals according to their subjective needs and expectations of difficulties functioning may be driven by personal and contextual factors [43]. An in-depth qualitative study could explore how individuals with different levels of VI understand their functional ability, and how their social and contextual circumstances influence that understanding. Further analysis of datasets containing clinical and self-reported measures of disability may also help identify determinants of non-agreement between these measures.

Further to the above, this study is subject to several limitations that are important to consider when interpreting the results. First, the cross-sectional design makes it impossible to know the directionality of relationships between VI and functional and mental health difficulties. Further research using longitudinal datasets would be necessary to answer questions of temporality and causality. Second, our dataset highlights important limitations with regards to ascertaining disability through self-reporting functional difficulties. Study participants clinically assessed to be blind and severely visually impaired, significantly under reported difficulties seeing, possibly by around 50%. Assuming participants also underreported functional difficulties in other domains, the overall prevalence of disability may be significantly underestimated and differences between individuals with and without functional difficulties could be underestimated. Consistently observed low specificity of ‘a lot of difficulty’ or’ cannot do at all’ compared with clinical measures indicate a need to explore the inclusion of ‘some difficulties’ in the definition of disability [41]. Third, the study area may not be representative of Uganda, and the results may not be generalisable beyond this population. The restriction of the study area to just those districts of Karamoja least affected by conflict may introduce bias into the datasets. Individuals living in the less secure districts may experience greater barriers to accessing eye care and are likely to have lower coverage than study participants. Similar studies in other parts of the country will be important to draw conclusions about the effect of disability on older people’s health in Uganda overall. Finally, access to healthcare is a multidimensional concept, going beyond coverage, and we did not seek to assess components of quality or affordability.

## Conclusion

This study provides important evidence about differences in VI and access to eye health services in a low-resource setting in northern Uganda. Prevalence of disability and multimorbidity is high and access to eye health services for all remains low. Further exploration of the factors that influence self-reporting functional difficulties in different contexts would support improved interpretation of such data.

## Supporting information

S1 Checklist Inclusivity in global research questionnaire. (DOCX)

S1 Table Distribution of domains of difficulties: Individuals reporting having a lot of difficulty or cannot do at all, or having a lot of anxiety/ depression on a daily basis, prevalence ratios adjusted for age. PR reference: No difficulties.
(DOCX)

S2 Table Associations between access to eye health services and a range of factors: Sensitivity analysis: Scenario when all blind and severely visually impaired individuals are categorised as having a functional difficulty. * p<0.05, ** p<0.01, *** p<0.001; ^†^ adjusted for gender only, ^‡^ adjusted for age only.
(DOCX)

## References

[1] He W, Aboderin I, Adjaye-Gbewonyo D. Africa Aging: 2020. Washington, DC: U.S. Census Bureau, 2020.

[2] Prince M, Wu F, Guo Y, Gutierrez-Robledo L, O’Donnell M, Sullivan R, et al. The burden of disease in older people and implications for health policy and practice. The Lancet. 2015;385(9967):549–62. doi: 10.1016/S0140-6736(14)61347-7 25468153

[3] Chatterji S, Byles J, Cutler D, Seeman T, Verdes E. Health, functioning, and disability in older adults—present status and future implications. The Lancet. 2015;385(9967):563–75. doi: 10.1016/S0140-6736(14)61462-8 25468158 PMC4882096

[4] Aboderin I, Beard J. Older people’s health in sub-Saharan Africa. The Lancet. 2015 2015/02/14/;385(9968):e9–e11. doi: 10.1016/S0140-6736(14)61602-0 25468150

[5] World Health Organization. World report on ageing and health: World Health Organization; 2015.

[6] World Health Organization. Global report on health equity for persons with disabilities: World Health Organization; 2022.

[7] Kuper H, Heydt P. The Missing Billion: Access to Health Services for 1 Billion People with Disabilities. 2019.

[8] Uganda Bureau of Statistics. Uganda Functional Difficulties Survey 2017. Kampala, Uganda: 2018.

[9] Statistics South Africa. Census 2022. Statistical Release P0301.4. Republic of South Africa: 2023.

[10] United Nations, Department of Economic and Social Affairs, Population Division. World Population Prospects 2022, Online Edition. 2022; Available from: https://population.un.org/wpp/Download/Standard/Population/.

[11] World Health Organisation. World Report on Disability. World Report on Disability 2011. Geneva: World Health Organization; 2011.

[12] Hashemi G, Wickenden M, Bright T, Kuper H. Barriers to accessing primary healthcare services for people with disabilities in low and middle-income countries, a Meta-synthesis of qualitative studies. Disability and Rehabilitation. 2020:1–14. doi: 10.1080/09638288.2020.1817984 32956610

[13] Ganle J, Baatiema L, Quansah R, Danso-Appiah A. Barriers facing persons with disability in accessing sexual and reproductive health services in sub-Saharan Africa: A systematic review. PLoS ONE. 2020 2020;15(10). doi: 10.1371/journal.pone.0238585 33044966 PMC7549766

[14] Casebolt M. Barriers to reproductive health services for women with disability in low- and middle-income countries: A review of the literature. Sexual & Reproductive Healthcare. 2020 2020/06/01/;24:100485. doi: 10.1016/j.srhc.2020.100485 32036280

[15] Horenstein A, Heimberg R. Anxiety disorders and healthcare utilization: A systematic review. Clinical Psychology Review. 2020 2020/11/01/;81:101894. doi: 10.1016/j.cpr.2020.101894 32818687

[16] Bechange S, Jolley E, Tobi P, Mailu E, Sentongo J, Chulu T, et al. Understanding patient health-seeking behaviour to optimise the uptake of cataract surgery in rural Kenya, Zambia and Uganda: findings from a multisite qualitative study. International Health. 2022 2022;14:I57–I63. doi: 10.1093/inthealth/ihab061 34581785 PMC8986356

[17] Burton M, Ramke J, Marques P, Bourne R, Congdon N, Jones I, et al. The Lancet Global Health Commission on Global Eye Health: vision beyond 2020. The Lancet Global Health. 2021;9(4):e489–e551. doi: 10.1016/S2214-109X(20)30488-5 33607016 PMC7966694

[18] Mushumbusi E, Buchan J, Mactaggart I, Macleod D, Foster A. A Systematic Review of the Proportion of Blindness in the Population 50 Years and Older from Total Population-Based Surveys of Blindness and Visual Impairment. Ophthalmic Epidemiol. 2022 Apr;29(2):164–70. doi: 10.1080/09286586.2021.1918176 33944649

[19] Kuper H, Polack S, Limburg H. Rapid assessment of avoidable blindness. Community Eye Health. 2006;19(60):68–9. 17515970 PMC1871676

[20] Uganda Bureau of Statistics (UBOS). Revised Subcounty Population 2015–2030. 2022.

[21] Kirkwood B, Sterne J. Essential medical statistics: John Wiley & Sons; 2010.

[22] Jolley E, Buttan S, Engels T, Gillani M, Jadoon M, Kabona G, et al. Prevalence of Visual Impairment and Coverage of Cataract Surgical Services: Associations with Sex, Disability, and Economic Status in Five Diverse Sites. Ophthalmic Epidemiol. 2020:1–9. 32449411 10.1080/09286586.2020.1768553

[23] Dimagi Inc. CommCare.

[24] World Health Organisation (WHO). ICD-11 for Mortality and Morbidity Statistics. 2018; Available from: https://icd.who.int/browse11/.

[25] Washington Group on Disability Statistics. The Washington Group Short Set on Functioning–Enhanced (WG-SS Enhanced). 2020 [May 10 2021]; Available from: https://www.washingtongroup-disability.com/question-sets/wg-short-set-on-functioning-–-enhanced-wg-ss-enhanced

[26] ESRI. ArcGIS. Pro ed2020.

[27] Statacorp LLC. Stata. 15.1 ed2018.

[28] Zou G. A Modified Poisson Regression Approach to Prospective Studies with Binary Data. American Journal of Epidemiology. 2004;159(7):702–6. doi: 10.1093/aje/kwh090 15033648

[29] Ministry of Gender Labour and Social Development, Uganda Bureau of Statistics. Situational analysis of persons with disabilities in Uganda. 2020.

[30] Wandera S, Ntozi J, Kwagala B. Prevalence and correlates of disability among older Ugandans: evidence from the Uganda National Household Survey. Glob Health Action. 2014;7:25686. doi: 10.3402/gha.v7.25686 25413721 PMC4238972

[31] Banerjee S. Multimorbidity—older adults need health care that can count past one. The Lancet. 2015;385(9968):587–9. doi: 10.1016/S0140-6736(14)61596-8 25468155

[32] Uganda Bureau of Statistics. Multi-dimensional Poverty Index for Uganda. Kampala: UBOS, 2022.

[33] Groce N, Kett M. The disability and development gap. Leonard Cheshire Disability and Inclusive Development Centre Working Paper Series. 2013 (21).

[34] Lewallen S, Mousa A, Bassett K, Courtright P. Cataract surgical coverage remains lower in women. Br J Ophthalmol. 2009;93(3):295–8. doi: 10.1136/bjo.2008.140301 19091848

[35] Mailu E, Virendrakumar B, Bechange S, Jolley E, Schmidt E. Factors associated with the uptake of cataract surgery and interventions to improve uptake in low- and middle-income countries: A systematic review. PLoS One. 2020;15(7):e0235699. doi: 10.1371/journal.pone.0235699 32645065 PMC7347115

[36] McCormick I, Butcher R, Evans J, Mactaggart I, Limburg H, Jolley E, et al. Effective cataract surgical coverage in adults aged 50 years and older: estimates from population-based surveys in 55 countries. Lancet Global Health. 2022 2022;10(12):e1744–e53. doi: 10.1016/S2214-109X(22)00419-3 36240806 PMC7618287

[37] Geneau R, Massae P, Courtright P, Lewallen S. Using qualitative methods to understand the determinants of patients’ willingness to pay for cataract surgery: A study in Tanzania. Social Science & Medicine. 2008 2008/02/01/;66(3):558–68. doi: 10.1016/j.socscimed.2007.09.016 18006201

[38] Aboobaker S, Courtright P. Barriers to cataract surgery in Africa: a systematic review. Middle East Afr J Ophthalmol. 2016;23(1):145. doi: 10.4103/0974-9233.164615 26957856 PMC4759895

[39] World Health Organization. World report on vision. Geneva: 2019.

[40] Rotenberg S, Rodríguez-Gatta D, Wahedi A, Loo R, McFadden E, Ryan S. Disability training for health workers: A global evidence synthesis. Disability and Health Journal. 2022 2022/04/01/;15(2):101260. doi: 10.1016/j.dhjo.2021.101260 35090840

[41] Boggs D, Kuper H, Mactaggart I, Bright T, Murthy G, Hydara A, et al. Exploring the Use of Washington Group Questions to Identify People with Clinical Impairments Who Need Services including Assistive Products: Results from Five Population-Based Surveys. International Journal of Environmental Research and Public Health. 2022;19(7):4304. doi: 10.3390/ijerph19074304 35409984 PMC8998283

[42] Mactaggart I, Kuper H, Murthy G, Oye J, Polack S. Measuring Disability in Population Based Surveys: The Interrelationship between Clinical Impairments and Reported Functional Limitations in Cameroon and India. PLOS ONE. 2016;11(10):e0164470. doi: 10.1371/journal.pone.0164470 27741320 PMC5065175

[43] Cockburn L, Roberts J, Lee S, Nganji J, Ho N, Kuntjoro A, et al. Considerations when asking about “disability” in disability inclusive research. Disability and Rehabilitation. 2023:1–20. 10.1080/09638288.2023.2291732 38095550

