# Supplementary material for: Differences in need for and access to eye health services between older people with and without disability: A cross-sectional survey in four districts of northern Uganda
Source: PLOS Glob Public Health. 2024 Sep 10;4(9):e0003645. doi: 10.1371/journal.pgph.0003645 (PMC11386432; doi:10.1371/journal.pgph.0003645)
Supplement: S1 Table — PR reference: No difficulties. (DOCX) [file pgph.0003645.s002.docx]

|  | **Male** |  | **Female** |  |  |  |
| --- | --- | --- | --- | --- | --- | --- |
|  | n | % | n | % | Age adjusted prevalence ratio | 95%CI |
| Seeing | 55 | 5.0 | 154 | 7.5 | 1.37 | 0.98-1.93 |
| Hearing | 23 | 2.1 | 65 | 3.2 | 1.42 | 0.87-2.31 |
| Walking | 33 | 3.0 | 106 | 5.2 | 1.56 | 1.04-2.36 |
| Communicating | 11 | 1.0 | 45 | 2.2 | 1.96 | 1.06-3.63 |
| Remembering or concentrating | 10 | 0.9 | 58 | 2.8 | 2.83 | 1.39-5.76 |
| Self-care | 7 | 0.6 | 27 | 1.3 | 1.85 | 0.74-4.65 |
| **Any functional difficulty (all six domains)*** | **106** | **9.6** | **246** | **12.0** | **1.16** | **0.92-1.45** |
| **Other functional difficulty (excludes seeing domain)*** | **51** | **4.6** | **92** | **4.5** | **0.98** | **0.86-1.12** |
| Anxiety | 91 | 8.2 | 292 | 14.3 | 1.71 | 1.31-2.23 |
| Depression | 39 | 3.5 | 156 | 7.6 | 2.10 | 1.36-3.25 |
| **Anxiety and/ or depression** | **112** | **10.1** | **326** | **15.9** | **1.54** | **1.21-1.97** |
| **Total any functional difficulty and/ or anxiety and/ or depression** | **198** | **17.9** | **488** | **23.8** | **1.28** | **1.06-1.54** |
| **Total other functional difficulty and/ or anxiety and/ or depression (excludes seeing domain)*** | **143** | **12.9** | **334** | **16.3** | **1.10** | **1.01-1.20** |
| **Multiple domains: difficulties functioning in more than one domain*** | **70** | **6.3** | **267** | **13.0** | **1.23** | **1.13-1.34** |
|  |  |  |  |  |  |  |
