# Supplementary material for: Differences in need for and access to eye health services between older people with and without disability: A cross-sectional survey in four districts of northern Uganda
Source: PLOS Glob Public Health. 2024 Sep 10;4(9):e0003645. doi: 10.1371/journal.pgph.0003645 (PMC11386432; doi:10.1371/journal.pgph.0003645)
Supplement: S2 Table — * p<0.05, ** p<0.01, *** p<0.001; † adjusted for gender only, ‡ adjusted for age only. (DOCX) [file pgph.0003645.s003.docx]

|  |  | **Models adjusted for age and gender only** | | | | **Multivariate models** | | | |
| --- | --- | --- | --- | --- | --- | --- | --- | --- | --- |
|  |  | **Operated cataract** | | **Operated trachoma** | | **Operated cataract** | | **Operated trachoma** | |
|  |  | **RR** | **95%CI** | **RR** | **95%CI** | **ARR** | **95%CI** | **ARR** | **95%CI** |
| Functional difficulties (ref no difficulties) | Seeing | 0.61 | 0.44-0.85** | 1.07 | 0.75-1.52 | 0.65 | 0.47-0.90** |  |  |
|  | Other | 1.30 | 0.95-1.79 | 1.35 | 0.73-2.51 | 1.19 | 0.84-1.69 |  |  |
| Mental health difficulties (ref no difficulties) |  | 0.77 | 0.57-1.04 | 1.15 | 0.77-1.72 |  |  |  |  |
| Age (ref 50-59) ^†^ | 60-69 | 1.20 | 0.49-2.92 | 1.26 | 0.63-2.53 | 1.32 | 0.60-2.89 | 1.21 | 0.64-2.27 |
|  | 70-79 | 1.91 | 0.86-4.24 | 1.47 | 0.80-2.70 | 1.81 | 0.91-3.61 | 1.23 | 0.71-2.14 |
|  | 80+ | 1.76 | 0.75-4.14 | 1.24 | 0.64-2.38 | 1.67 | 0.79-3.56 | 1.09 | 0.59-2.00 |
| Gender (ref male) ^‡^ | Female | 0.70 | 0.54-0.90* | 1.06 | 0.67-1.68 | 0.77 | 0.61-0.98* |  |  |
| Poverty (ref no regular income) | Regular income | 2.03 | 1.51-2.73*** | 1.69 | 1.25-2.27** | 1.67 | 1.08-2.57* | 1.59 | 1.02-2.49* |
| Marital status (ref married) | Widowed, never married or separated | 1.04 | 0.83-1.29 | 1.41 | 1.04-1.91* |  |  | 1.29 | 0.93-1.79 |
| Children living near you (ref 5+ children) | <5 | 1.07 | 0.90-1.27 | 1.06 | 0.84-1.33 |  |  |  |  |
| Household size (ref 5+) | <5 | 1.07 | 0.79-1.45 | 0.87 | 0.54-1.38 |  |  |  |  |
| Distance from health centre with ophthalmologist (per 10 km) | 10 km | 1.06 | 1.00-1.11* | 1.05 | 1.00-1.11* | 1.03 | 0.95-1.11 | 1.00 | 0.94-1.07 |
| Distance from any health centre (per 10 km) | 10 km | 0.92 | 0.86-0.99* | 0.93 | 0.78-1.10 | 0.93 | 0.84-1.03 |  |  |
| Accessed healthcare last time it was needed (ref yes) | No | 0.74 | 0.48-1.13 | 1.17 | 0.80-1.71 |  |  |  |  |
